# Postoperative Delirium and Dementia in Patients Undergoing Cardiac Surgery: A Review of Randomized Controlled Trials

**DOI:** 10.31661/gmj.v12i.3045

**Published:** 2023-08-23

**Authors:** Venus Shahabi Raberi, Morteza Solati Kooshk Qazi, Ali Zolfi gol, Rahil GhorbaniNia, Ozra Kahourian, Reza Faramarz Zadeh

**Affiliations:** ^1^ Seyed-Al-Shohada cardiology Hospital, Urmia University of Medical Sciences, Urmia, Iran; ^2^ School of Nursing and Midwifery, Shiraz University of Medical Sciences, Shiraz, Iran; ^3^ Department of Pediatric Cardiology, Shahid Motahari Hospital, Hospital, Urmia University of Medical Sciences, Urmia, Iran; ^4^ Noncommunicable Diseases Research Center, Bam University of Medical Sciences, Bam, Iran

**Keywords:** Dementia, Postoperative Complications, Cardiac Surgery

## Abstract

Delirium and dementia are considered to be the most significant postoperative neurocognitive complications in patients undergoing cardiac surgery, particularly those aged 60 years and older, which reduces the post-surgery quality of life, prolongs hospitalization, increases costs, and elevated the rates of mortality. Nevertheless, the etiology, risk factors, and predictive biomarkers, have not been well elucidated particularly, in patients with unmanifested underline cognitive impairments. The present study aimed to review the findings on the etiology, factors increasing the risk of incidence, and predictive biomarkers of postoperative delirium and dementia after cardiac surgery, and to describe the suggested pharmacological and non-pharmacological interventions.

## Introduction

Postoperative cognitive decline (POCD) is a common sub-clinical condition following cardiac and non-cardiac surgery [1][2]. POCD is widely considered to be the most prominent in people over the age of 65 [3], and as the population all over the world is aging at an unprecedented rate, POCD has become more common [4]. Patients with POCD will generally experience a variety of neurological symptoms including anxiety, memory impairment, personality change, and mental confusion all of which lead to increased medical costs, reduced life independence, and are associated with mortality [4]. Currently, several types of POCD-related disorders have been listed, of which delirium and dementia are the most well-known. In addition, the cognitive decline caused by anesthesia or surgery could be followed by delirium or dementia [5][6].

Delirium is considered an acute fluctuating alteration in mental state that manifests as a disturbance in consciousness and cognitive function. There is a suggestion that delirium has a high incidence rate in hospitalized patients and is followed by increased rates of morbidity and mortality, and prolonged residence in the intensive care unit (ICU) and the hospital, thereby higher costs for both the patient and the government [7]. Postoperative delirium (POD) is described as a common complication after cardiac surgery [8] in which geriatric patients, cardiac surgery patients, and patients hospitalized in ICU are highly at risk of developing POD [9][10]. POD patients are widely believed to have longer hospital stays, a higher risk of dementia, and a higher risk of mortality [11].

Delirium can progress to dementia, which is characterized by the loss of cognitive functions including remembering, thinking, and reasoning. The development of dementia after delirium is of great concern to both third leading cause the patient and public health since the significant morbidity, mortality, and caregiver burden associated with dementia have been documented [12][13].

Importantly, dementia has recently ranked as the third leading cause of death in the United States [14]. Annually, more than 47 million people suffer from dementia which is equivalent to 5% of the world’s elderly population [15].

More importantly, it is expected that the incidence of dementia will increase substantially in the following years due to an aging population [14].

Recent studies have demonstrated that cardiac surgery could be associated with increased levels of biomarkers related to neurodegeneration including neurofilament light chain and Tau [16][17][18]. Despite the mentioned importance of postoperative delirium and dementia after cardiac surgery, the etiology of its occurrence has not been elucidated, and contradictory propositions have been hypothesized. Therefore, the present study attempted to clarify the main causes of postoperative delirium and dementia after cardiac surgery and compensatory pre-, intra-, and post-operative proceedings to prevent/reduce its incidence by reviewing the randomized controlled trials.

Cardiac Surgery Leads to Delirium and Dementia

In general, the pre-surgery cognitive state, the application of anesthetics, procedures and injuries during surgery, along with induced inflammation and stress are the most important assumptions about the etiology of cardiac surgery-related cognitive impairment. However, contradictions still exist in this regard, as new findings consistently weaken certain hypotheses and strengthen others. Thereby, the current study will review randomized controlled trials to analyze the assumptions about the etiology of postoperative delirium and dementia in patients undergoing cardiac surgery and then will assess the necessary proceedings to confront it.

Initially, it was thought that anesthetics used beforecardiac surgery would be the most important cause of POCD and subsequent POD and post-operative dementia, although subsequent studies have shown that the surgical process is associated with inflammation, oxidative stress, hypoxemia, and damage to the blood-brain barrier (BBB) as a causative agent, too [5][6]. It seems that patients who have an underlying neurodegenerative complication, although not manifested yet, are more susceptible to experiencing a condition known as a second hit. In fact, second hits are able to accelerate the process of neurodegeneration by the induction of cognitive decline after cardiac surgery [19]. The measurement of pre-and post-surgery levels of a critical biomarker of Alzheimer’s disease, cerebrospinal fluid (CSF) amyloid-β42 (Aβ42), in patients with POCD can be considered the most important finding supporting this hypothesis [20].

Induction of inflammation can be described as a common feature of surgical procedures, especially cardiac surgery, which can be assumed as a bridge between surgery and POCD, POD, and postoperative dementia due to the involvement of inflammation in the development of neurodegenerative disorders [21][22][23]. C-reactive protein (CRP), for example, is an appropriate marker of inflammation in patients with delirium particularly those undergoing surgery [24]. Moreover, a dramatic elevation in the levels of inflammatory markers including interleukin-6 (IL-6), fibroblast growth factor 21 (FGF21) and 23, and CC-chemokine ligand 2 (CCL2) is reported [25][26][27]. Furthermore, glial fibrillary acidic protein (GFAP) is a neuroinflammatory marker that could represent the link between inflammation and neurodegenerative complications [28].

Risk Factors of Postoperative Delirium and Dementia after Cardiac Surgery

The determination of the association between the operative procedure, anesthetics, inflammation, and so forth, also postoperative neurological complications could benefit the understanding of the etiology of POD and postoperative dementia, however, it requires comprehensive studies. Indeed, multidisciplinary studies are needed to explore the overlap between preexisting cognitive impairment and POCD, POD, and postoperative dementia in the fields of anesthesia, surgery, and old age psychiatry and neurology. The intradisciplinary alignment of the terminology and diagnostic criteria may improve our understanding of the etiology, biomarkers, and preventive strategies [5][29].

Understanding the cause of cognitive impairment after cardiac surgery can lead to the determination of relevant biomarkers. As a result, the potential biomarkers might estimate the probability of POD and the development of POD to dementia in patients undergoing cardiac surgery before and after the procedure [30][31].

Although the relevant biomarkers have not been fully understood, it is documented that elder hospitalized patients with delirium represent a remarkable 12-fold increase in developing dementia, hence delirium could be considered a reliable marker of postoperative dementia [32][33]. Along with that, several attempts have recently been made to identify risk factors associated with POD and dementia after cardiac surgery. Although scientists believed that underlying cognitive impairments before surgery, whose symptoms did not manifest were responsible for POD and dementia after cardiac surgery, Lewis et al. reported evidence against this belief [34].

By examining 320 patients who underwent cardiac surgery, it was found that 15.6% of patients had depression before surgery and 13.4% of patients experienced depression after surgery. Interestingly, preoperative depression was mainly associated with increased anxiety and decreased self-ratings on several quality-of-life domains, while the experience of postoperative depression four weeks after surgery was associated with neurocognitive complications including as poor memory, attention, processing speed, verbal fluency, and fine motor speed [34]. As a result, it can be assumed that although preoperative depression does not cause further neurocognitive disorders, postoperative depression could be related to several cognitive impairments, hence following the patients’ condition in terms of depression after surgery can be a preventive approach to confront delirium and dementia. In addition, a 5-year follow-up study of 114 elderly patients aged 70 and over who underwent cardiac surgery showed that preoperative mild cognitive impairment might be a risk factor for delirium and dementia after cardiac surgery [35].

Also, 87% of the patients who had experienced dementia within 5 years after surgery had experienced POD, too [35]. Therefore, POD can be considered a potential risk factor for the development of dementia after cardiac surgery. In fact, assessment of the preoperative cognitive function in elderly patients undergoing cardiac surgery should be screened. Moreover, patients who have experienced POD should be followed up to enable the early detection of dementia symptoms and to prevent the subsequent drastic consequences. In addition to pre- and postoperative cognitive status, a number of cardiac and inflammatory biomarkers can be considered risk factors for POD and subsequent dementia. CRP, for example, is considered the most reliable preoperative biomarker for the prediction of POD in patients undergoing noncardiac surgery [36]. Similarly, the increased levels of IL-2 and TNF-α were significantly associated with POD in patients undergoing coronary artery bypass grafting, and more importantly, researchers have provided cutoff scores for increased risk [37].

In patients undergoing cardiac surgery, particularly those experiencing cardiopulmonary bypass, a systematic stress and subsequently systematic inflammatory response occurs accompanied by elevated levels of inflammatory markers including cytokines and chemokines. Also that could contribute to devastating processes including the dysfunction of endothelial and the disruption of BBB [38]. Consequently, these processes could be followed by the susceptibility of the brain to neuronal damage by neuroinflammatory mediators and the activation of microglia leading to the development of POD [39]. In addition, plasma levels of several neurotransmitters including reduced cholinesterase and increased dopamine could be considered biomarkers of POD [39][40]. On the contrary, Wiberg et al., by studying 193 patients undergoing coronary artery bypass grafting and/or aortic valve replacement, demonstrated that higher (70-80 mmHg) or lower (40-50 mmHg) scores of mean arterial pressure during cardiopulmonary bypass had no significant association with cerebral injury biomarkers including neuron-specific enolase, tau, neurofilament light, and the glial marker known as glial fibrillary acidic protein [41]. In this sense, it is encouraged to conduct further randomized controlled trials measuring pre, inter, and postoperative heart-related markers and assess their association with biomarkers of cerebral injury and outcomes of pre and post surgery brain imaging tests.

Preventive Interventions for Delirium and Dementia after Cardiac Surgery

Although the etiology, risk factors, and biomarkers of delirium and dementia after cardiac surgery are still not fully understood, interventions have been conducted in several randomized controlled trials to prevent postoperative cognitive impairment in patients undergoing cardiac surgery. In addition to addressing undesirable neurocognitive consequences after surgery, such interventions can elucidate the causes of occurrence and make available risk factors. The randomized controlled trials conducted so far have attempted to prevent delirium and dementia after heart surgery through two types of interventions, including pharmacological interventions and non-pharmacological (operative) interventions reviewed below.

Pharmacological Interventions

Anesthesia and cardiac surgery procedures have been hypothesized as the main possible causes of cognitive post surgery impairments due to inducing systematic inflammation and oxidative stress. Therefore, in several studies, researchers have attempted to reduce or eliminate the neurocognitive adverse effects of surgery through pharmacological interventions capable of reducing inflammation and stress. Ketamine is an anesthetic with anti-inflammatory properties that can reduce POD in animal studies [42][43]. Moreover, a human study revealed that adding ketamine to routine anesthetics could reduce POD from 31% in the placebo group to 3% [44]. A randomized controlled trial on patients aged 65 years and older undergoing cardiac surgery with cardiopulmonary bypass demonstrated that during cardiopulmonary bypass the infusion of ketamine (31.25%) significantly reduced 24-h POD after surgery compared to propofol (56.25%) [45].

The application of sedative compounds is another pharmacological intervention that has been studied to reduce or eliminate neurocognitive consequences. Dexmedetomidine, an α2 adrenoceptor agonist, is frequently administered to patients in the intensive care unit due to its sedative and analgesic properties [46][47]. Perioperative administration of dexmedetomidine was accompanied by desired outcomes including reduction of opioid utilization, improvement of postoperative analgesia, and suppression of inflammation all of which are considered possible causes of cognitive impairment [48][49]. In addition, postoperative administration of dexmedetomidine led to a lower risk of suffering from mental complaints in patients who underwent cardiac surgery [50].

A single-center, double-blind, randomized controlled clinical trial on 508 patients undergoing cardiac surgery, of which 251 people received dexmedetomidine and 257 participated in the placebo group, revealed that psychological impairment could be significantly reduced in the dexmedetomidine group relative to the placebo group [51]. Similarly, another single-blinded, prospective, randomized controlled trial in 183 elder patients 60 years or older undergoing cardiac surgery, of which 91 patients in the dexmedetomidine group and others received propofol, demonstrated that this sedative agent compared to propofol reduced the incidence (17.5% compared to 31.5%), delayed onset (day 2 versus day 1), and shortened duration of POD (2 days versus 3 days) [52].

Moreover, the participation of 46 patients who underwent coronary artery bypass graft surgery participated in a randomized controlled trial, suggested that dexmedetomidine compared to typical anesthesia could increase the levels of neural protective biomarkers including matrix metalloproteinase-12 and myelin basic protein [53]. However, a higher rate of hypotension was reported as an adverse effect of dexmedetomidine administration requiring further studies to elucidate other possible side effects [51].

Piracetam is a derivative of the neurotransmitter γ-aminobutyric acid with cerebroprotective properties that its administration leads to better cognitive function in patients undergoing coronary artery bypass surgery and reduces the early postoperative substantial decline of neuropsychological performances [54]. Melatonin is a pineal gland hormone thought to be important in sleep/wake regulation which has been used in a wide range of studies from neurological disorders to environmental improvements [55][56].

Several studies over the past decade have shown that the administration of this hormone can significantly reduce POD and post-surgery sleep/wake complications [55]. However, a randomized double-blind controlled clinical trial 7 days of treatment with melatonin starting 2 days before the surgery did not support the prophylactic application of this hormone to prevent POD [57].

Similarly, a double-blind, randomized, controlled study showed that dietary melatonin therapy in patients with mild cognitive impairment can significantly increase hippocampal volume and significantly reduce CSF T-tau protein levels [58].

Because mild cognitive impairment is a transitory state to dementia, while delirium is a reliable risk factor for progression to dementia, administration of melatonin is associated with cardiac surgery, although no confirmatory studies have been performed. Insulin is another hormone its administration at normoglycemia during cardiac surgery may lead to the prevention of short- and long-term memory decline postoperatively [59].

A randomized, double-blind controlled trial revealed that the administration of insulin intranasally during cardiac surgery in patients with type 2 diabetes does not cause hypoglycemia which could be important for neural cells [60].

Non-pharmacological Interventions

Non-pharmacological interventions to reduce delirium and dementia after cardiac surgery can be divided into three categories: pre-, intra-, and post-surgery interventions. However, most of the interventions are related to the procedures during and after surgery.

Cognitive training is a well-studied peri and intraoperative intervention though to be able to durably improve cognitive reserve in POCD and POD and thereby could potentially reduce the risk of postoperative cognitive impairment in patients undergoing cardiac surgery. A randomized, single-center, controlled trial studied the perioperative cognitive training efficacy in 65 patients elderly aged 60-90. The findings showed that this program could significantly reduce the risk of POCD and POD although further studies were encouraged [61].

In addition, another randomized clinical trial on 60 elderly patients revealed that postoperative cognitive training could improve health-related quality of life and reduce cognitive failure three months after heart valve surgery [62]. Early mobilization was another strategy that Shirvani et al. introduced to reduce POD via a double-blind randomized clinical trial on 92 patients undergoing coronary artery bypass grafting [63].

Other interventions have been focused on cerebral oxygenation. A randomized controlled pilot study on 82 patients, older than 65, who underwent coronary artery bypass graft surgery on cardiopulmonary bypass revealed that the incidence of POD in the intervention group of patients, was significantly lower (2.4%) compared to controls (20%) in patients whose regional cerebral tissue desaturations of more than 15% of the pre-induction value or below 50% were avoided [64].

Similarly, the optimization of cerebral oxygenation and observance of intraoperative oxygen concentration are considered strategies for reducing the risk of postoperative impairment of cognitive ability in elderly patients undergoing cardiac surgery [65][66]. In addition, hemodilution during coronary artery bypass grafting using cardiopulmonary bypass (maintaining the hematocrit above 25% by transfusion of packed red blood cells), performing an aortic off-pump coronary artery bypass instead of conventional procedure, and the application of conservative strategies (coronary angiogram only if recurrent ischemia or heart failure) instead of invasive approaches (routine coronary angiogram) are among the most important interventions conducted to reduce the risk of delirium and dementia after heart surgery [67][68][69].

## Conclusion

Delirium and dementia are among the most important neurocognitive complications after cardiac surgery. However, the major causative factors are not fully understood, nor are the risk factors and biomarkers. The present study revealed that despite the valuable efforts during recent years, the etiology and definitive diagnosis of these complications, especially in elderly people who do not manifest symptoms of precognitive disorders, requires further investigations. In addition, pharmacological interventions including sedative agents or adjuvant anesthetics along with non-pharmacological interventions such as cognitive training, cerebral oxygenation, novel surgical approaches, and hemodilution can significantly reduce the risk of postoperative delirium and dementia.

## Conflict of Interest

The authors declare that there are no conflicts of interests.
